# Directionality theory and the origin of life

**DOI:** 10.1098/rsos.230623

**Published:** 2024-11-13

**Authors:** Lloyd A. Demetrius

**Affiliations:** ^1^ Department of Organismic and Evolutionary Biology, Harvard University Cambridge, Cambridge, MA 02138, USA

**Keywords:** self-organization, evolutionary entropy, RNA world

## Abstract

The origin of cellular life can be described in terms of the transition from inorganic matter to the emergence of cooperative assemblies of organic matter: DNA and proteins, capable of replication and metabolism. Directionality theory is a mathematical theory of the collective behaviour of networks of organic matter: activated macromolecules, cells and higher organisms. Evolutionary entropy, a generalization of the thermodynamic entropy of Boltzmann, is a statistical measure of the cooperativity of the biotic components. The cornerstone of Directionality theory is the *Entropic Principle of Evolution:* evolutionary entropy *increases* in systems driven by a stable energy source, and *decreases* in systems subject to a fluctuating energy source. This article invokes the Entropic Principle of Evolution—an extension to biological systems of the Second Law of Thermodynamics—to provide an adaptive rationale for the following sequence of transformations that define the emergence of cellular life: (i) the self‐assembly of activated macromolecules from inorganic matter; (ii) the emergence of an RNA world, defined by RNA molecules with catalytic and replicative properties; and (iii) the origin of cellular life, the integration of the three carbon-based polymers—DNA, proteins and lipids, to generate a metabolic and replicative unit.

## 1. Introduction

Cellular life, the fundamental unit of living organisms, consists of an elaborate set of self-reproducing chemical reactions. These reactions rely upon a large set of specific catalysts, encoded by a DNA genome. The metabolic and replicative components of cells are determined by three classes of carbon-based polymers:

1. *Amphiphiles.* These polymers form the boundary membranes of all cellular life.
2. *Proteins.* These biopolymers are synthesized by linking together amino acids using an external source of energy. A group of proteins, called enzymes, have the unique ability to act as catalysts.
3. *DNA.* The subunits of these polymers are nucleotides. These biopolymers encode and transmit genetic information.

Cellular life is a process in which informational and catalytic polymers interact to generate a biochemical network where information in the DNA directs the synthesis of proteins, and the catalytic activity of proteins regulates the synthesis of DNA.

Modern cellular systems are isothermal, chemical machines that appropriate chemical energy from the external environment and convert this energy into biological work. During growth, the cyclic system of polymers reproduces itself, whereas mutations in the DNA generate differences in the metabolic capacity of the daughter cells. Since cells vary in their capacity to appropriate and convert chemical energy into biomass, selection will occur, and the composition of the population will evolve.

These observations suggest that the problem regarding the origin of life can be described as follows: *what are the physico-chemical principles that underlie the transition from the abiotic molecules that define condensed matter to the integrated assembly of organic matter—DNA, RNA and proteins—that characterizes cellular life?*

The empirical studies begun by Harold Urey and Stanley Miller in 1953, have provided a conceptual template for addressing this problem. The Miller–Urey studies showed that organic molecules could be created from inorganic matter by environmental conditions, such as heat and electric discharge, without the mediation of enzymes. This discovery suggested that the transition from inorganic matter to activated biomolecules, the basic components of organic matter, can occur spontaneously, provided there exists an external source of energy.

However, the transition from the state of activated biomolecules to cellular life appeared intractable, due largely to the complexity of the processes that appeared to underlie the origin of proteins and DNA [1].

Empirical studies of the catalysis of metabolic reactions in modern cells have elucidated the transition from activated biomolecules to cellular life by delineating two kinds of catalytic mechanisms [2,3], namely:

- *Class I reactions*. These metabolic processes are catalysed by ribonucleoprotein complexes. These reactions are RNA-based and first evolved in cells that contained no protein or DNA.
- *Class II reactions*. The molecules that are involved in these systems are proteins with an elaborately folded catalytic surface. These molecules emerged relatively late in the evolutionary history of the cell.

The discovery by Cech [4] that RNA can act as both a catalyst and a template suggested that modern-day cells are the evolutionary derivatives of an RNA world in which all molecular catalysts were ribozymes.

RNA is a less effective information and catalytic polymer than the DNA and protein that perform these functions in modern cells. DNA is more stable and less susceptible to mutation than RNA. Proteins have more complex three-dimensional structures than RNA. These characteristic features of RNA have provided an evolutionary rationale for the existence of an RNA world, and its subsequent replacement by a system whose operational units are DNA and protein [2,5,6].

Accordingly, the transformation from the emergence of activated bio-molecules—carbon-based monomers—to the organization of cellular life can be understood in terms of the following transitions [2,7,8]:

- *The emergence of RNA as an informational polymer.* The support for this emergence derives from the capacity of RNA to perform with the help of metals, and other small molecules, reactions that exhibit template and catalytic activity.
- *The transition from RNA to protein.* RNA can fold in complex ways that endow the molecules with the catalytic activity associated with proteins. Proteins, however, are more effective catalysts.
- *The transition from RNA to DNA.* RNA can also fold to assume the double helix structure, which enables storage of genetic information. DNA, however, is more stable and less vulnerable to replication errors.

The pioneering investigations of Urey & Miller [9], and the recent discoveries regarding the RNA world, as reviewed in [2,5,10,11] have stimulated a large body of theoretical and empirical research concerning the biophysical and chemical processes that integrated the three carbon-based polymers, RNA, protein and DNA, to generate cellular life. Recent theoretical contributions (e.g. [12–16]) have focused on questions regarding the process of metabolic complexification, or template replication, as initiating the emergence of cellular life. These studies have contributed to the understanding of autocatalytic processes.

Several models have implicitly rejected the existence of an RNA world, and the subsequent emergence of proteins, as the more effective catalyst, and DNA as the more stable information carrier.

De Duve [17] replaced the RNA world with a model that describes how a proto-metabolic web may have emerged prior to the appearance of membranes and of cells. The computational studies of Kauffman [18] are based on models of interacting prebiotic precursors, and the generation of autocatalytic networks. Hartman [19] also rejects the hypothesis of an RNA world and postulates the origin and evolution of photosynthesis as the cardinal process that underlies the transition to organic matter and cellular life.

Greenwald *et al*. [20] contend that, while RNA may have been a critical element at certain stages in the emergence of life, there may have been less complex precursors. These authors have postulated a prebiotic world based on amyloidogenic peptides, and have provided some experimental evidence for the so-called ‘amyloid-world’ hypothesis.

A synergistic model, based on the concept of systems chemistry, proposes that the emergence of life is derived from the generation of a mixture of diverse classes of macromolecules. This strategy is illustrated in the work of Powner & Sutherland [21], in studies that show how chemical subsystems may engender nucleotides and lipids by means of molecular synergism.

Although systems chemistry is a theoretically viable methodology for synthesizing the molecules essential to early life, the dominant model of prebiotic chemistry, in view of its consistency with the cell’s evolutionary history, remains the RNA world hypothesis [2]. Studies are now focused on empirical and theoretical studies to elucidate the transition to cellular life in terms of the biochemical and biophysical properties of RNA [6,22,23].

The empirical investigations are concerned with the geological environments and the biochemical sequence of events that determine the transitions from inorganic matter to the assembly of the carbon-based polymers, DNA, RNA and proteins, which are the basic components of cellular life.

The theoretical studies aim to determine the general principles of collective behaviour that encode the transition from lower-level elements, such as DNA, RNA and proteins, to higher-level aggregates, such as protocells and cellular life.

### 1.1. Empirical considerations

The empirical aspects that define the emergence of cellular life can be reduced to an analysis of its geochemical origins, the assembly of its biochemical components, and the organization of the closed compartments in which the chemical reactions occur.

The geochemical factor pertains to the geological environment and the corresponding energy source that would enable the transition from inorganic matter to the supramolecular structures that characterize replication and metabolism in cells [11]. The geological environments that have been proposed are hydrothermal vents [24,25] and hot springs [26]. Hydrothermal vents, an aquatic energy source, constitute chemically reactive environments capable of driving the synthesis of reactive molecules like formaldehyde and hydrogen cyanide. Hot springs, a terrestrial energy source, derive their effect from the empirical fact that lipid-encapsulated polymers can be generated by hydration and dehydration cycles. The complementary nature of these models and their relevance for understanding the transition from inorganic components to biosynthetic precursors are reviewed in [27]

The biochemical factor concerns the existence of an RNA molecule that can function both as a polymerase with catalytic activity, and as a template with replicative properties. Empirical support for the existence of the autocatalytic property of RNA is described in [22] and reviewed in [7].

Horning & Joyce [23], in an effort towards reconstructing an RNA world, exploited the process of *in vitro* evolution to enhance, by variation and selection, the activity of an RNA polymerase ribozyme. The observation that the resulting polymerase has enhanced replicative and catalytic activity gives added support for the existence of an RNA world [28].

The stability of the RNA world is contingent on the capacity of cells to regulate the flow of nutrients from the external environment to its interior. This regulation is achieved by membranes, composed of amphipathic lipids, which act as barriers to ensure that molecules that are closely related are confined to the same region. Amphiphiles, such as short-chain fatty acids, can self-assemble into stable vesicles.

The cell membrane consists of three classes of amphipathic lipids: phospholipids, glycolipids and sterols. The amount of each class depends on the cell type. The regulatory function of the different types and their role in self-assembly is reviewed in [29].

These geochemical observations and biosynthetic studies give support to the proposition that the emergence of cellular life can be understood in terms of the following sequence of events [2,6,30]:

- the assembly of components of inorganic matter to form activated molecules,
- the polymerization of these monomers to form amphiphiles and RNA polymers,
- the emergence of localized compartments to confine the RNA polymers,
- the assembly of the RNA polymers into localized compartments: the generation of protocells, defined by RNA molecules that function as both information carriers and as catalysts,
- the transformation from the protocellular state to cellular life: the integration of the three carbon-based polymers, DNA, RNA and proteins, to generate a metabolic and replicative cellular unit.

### 1.2. Theoretical considerations

Matter has a hierarchical structure. The transition from atoms and molecules (the basic components of inorganic matter) to cellular life (the basic components of living matter) is characterized by transformations between hierarchies defined by large differences in energy and size of the individual components.

The emergence of inorganic matter can be described as a cooperative phenomenon, defined in terms of the collective behaviour and the dynamics of atoms and molecules.

The emergence of living matter —the origin of cellular life—can also be described as a cooperative phenomenon. Cooperativity, at this hierarchical level, is in terms of two related processes:

- the collective behaviour of inorganic matter to generate protocells,
- the integrative action of protocells to produce cellular life.

#### 1.2.1. Atoms and molecules to inorganic matter

The collective behaviour of atoms and molecules to generate the macroscopic phenomena that characterize inorganic matter - solids, liquids and gases, can be understood in terms of statistical thermodynamics.

A central notion of this model of collective behaviour is the distinction between a *microstate*—a condition in which the variables associated with the individual particles are specified—and a *macrostate*—which provides information regarding variables such as temperature, energy and pressure.

Boltzmann’s study of the collective behaviour of the atoms and molecules that comprise inorganic matter is based on the following assumptions:

I(a) the individual components are identical,
I(b) the number of components is very large,
I(c) the dynamics of the individual atoms is governed by the laws of classical mechanics.

Collective behaviour is based on a model that considers the molecules of the system as having discrete energies, ϵ, 2ϵ, 3ϵ,….

Let N denote the total number of molecules, $n_{k}$ denote the number with energy kϵ, and W denote the number of microstates associated with a given macrostate. A combinatorial argument shows that


(1.1) $$W=\left[\frac{N!}{n_{1}!n_{2}!\ldots n_{s}!}\right],$$

where


$$N=\sum_{\kappa =1}^{s}n_{\kappa }.$$

The Boltzmann entropy $S_{B}$ is defined by


(1.2) $$S_{B}=k_{B}\;\log W.$$

The scale factor $k_{B}$ is Boltzmann’s constant. The cornerstone of statistical thermodynamics pertains to the change in thermodynamic entropy induced by the dynamics of the individual molecules. This property is encoded in terms of the following tenet.

*The Second Law of Thermodynamics*. In systems that are isolated and closed to the input of energy and matter, thermodynamic entropy increases.

This law entails that the equilibrium states of systems evolving under conditions that are isolated and closed to the input of energy and matter are described by states that maximize thermodynamic entropy.

Boltzmann’s statistical theory of irreversible non-equilibrium behaviour in physical systems has been immensely successful in explaining the collective behaviour of the atoms and molecules that comprise inorganic matter. The analysis is based on fundamental assumptions regarding the homogeneity of the atoms and molecules, the number of components and the laws that describe the molecular interactions.

Biological systems, in sharp contrast to physical systems, are composed of hierarchical networks of organic matter: activated macromolecules, carbon-based polymers, organelles and protocells. The assumptions regarding homogeneity, size and interaction dynamics, which are invoked in studies of the collective behaviour of atoms and molecules, are not valid for biological systems. This fact is often ignored in various efforts to apply concepts such as thermodynamic entropy, and principles such as the Second Law to elucidate the emergence of cellular life.

#### 1.2.2. Inorganic matter to cellular life

The transition from inorganic matter to the integration of the three carbon-based polymers—DNA, protein and RNA—that define cellular life, involves a sequence of processes characterized by the following transitions:

- inorganic matter to activated macromolecules,
- activated macromolecules to informational polymers and amphiphiles—the boundary membranes of all cellular life,
- protocells to cellular life.

The analysis of the collective behaviour of the components that define a biological network has its origin in Directionality theory, a general theory of the evolutionary dynamics of structured populations [31–33].

A population consists of a group of individual components who cooperate through the spreading and sharing of energy. The population can be formally represented in terms of a strongly connected, directed graph. The nodes of the graph describe the individual components. The links between the nodes represent the transfer of energy between the components.

The evolutionary dynamics of the population is based on the following assumptions:

II(a) the individual components are distinct,
II(b) the number of components is finite, and
II(c) dynamical changes in cooperativity are induced by the process of variation and natural selection.

In this model of evolutionary dynamics, cooperativity is defined in terms of the number of pathways of energy flow between the components that define the network. An analytic description of cooperativity is given by the expression, H=S/T. The functions S, called evolutionary entropy, and T, the cycle time, are given by


(1.3) $$S=-\sum_{\tilde{\alpha }\in C_{\alpha }}p_{\tilde{\alpha }}\;\log p_{\tilde{\alpha }}\;;\;T=\sum_{\tilde{\alpha }\in C_{\alpha }}|\tilde{\alpha }|p_{\tilde{\alpha }}.$$

The object $C_{\alpha }$ describes the set of pathways of the directed graph that begins at a node α and returns to α without passing through α en route. The function $p_{\tilde{\alpha }}$ is the probability of the cyclic pathway, $\tilde{\alpha }$.

The parameter *H* = *S*/*T*, which we also call evolutionary entropy, describes the rate at which the population converts the chemical energy of resources into biological work. The statistical parameter also describes robustness or stability, that is the rate at which a system returns to its steady state after a perturbation of its components.

The cornerstone of Directionality theory is a set of principles that describe changes in evolutionary entropy, due to the emergence of a variant subpopulation, and competition between the variant subpopulation and the resident.

The adaptive dynamics of evolutionary entropy due to natural selection is described by the following rule:

*The Entropic Principle of Evolution*: evolutionary entropy increases in systems driven by a stable energy source, and decreases in systems regulated by a fluctuating energy source.

The Entropic Principle of Evolution entails that the steady states of biological systems are contingent on the robustness of the external energy source, and determined by extremal states of evolutionary entropy—a maximization of evolutionary entropy, when the energy source is stable; a minimization of evolutionary entropy, when the energy source is unstable.

The carbon-based polymers RNA, protein and DNA have all evolved under different but stable resource conditions. Accordingly, their molecular configurations will be determined by the maximization of evolutionary entropy.

### 1.3. Implications and predictions

RNA is single stranded and can assume a variety of secondary structures depending on the sequence. This polymer has both catalytic and replicative properties. Proteins have an alpha-helical structure and are kinetically stable. They are effective catalysts and chemically active molecules, whose chemically variable parts are located on the outside of the protein. DNA has a double helical structure. These molecules encode the genetic information. The polymers are chemically stable elements, and the information-containing units, the nucleotide bases, are located at the core of the helix [2].

According to the RNA world [2,4,9], the transition from a single-stranded RNA, with catalytic and replicative properties, to the integrative activity of the three carbon-based polymers, RNA, protein and DNA, occurred at different stages in the evolutionary history of the cell, and according to the following transitions:

- autocatalytic RNA to a system involving RNA and proteins
- RNA–protein complex to an integrated assembly consisting of RNA, protein and DNA.

We will invoke the *Entropic Principle of Evolution* and the evolutionary history of the cell to explain the differences in stability of the three polymers. We will also exploit the principle to elucidate differences in the aetiology of familial and sporadic forms of age-related diseases.

#### 1.3.1. Proteins and DNA: stability

According to the Entropic Principle of Evolution, the structure of proteins and DNA will be characterized by molecular configurations that maximize evolutionary entropy, contingent on the external environment.

Proteins perform a variety of functions, catalysing chemical reactions, regulating genes, maintaining cellular homeostasis. These will implicate various modes of interaction with the chemical and physical environment. Accordingly, the maximization of evolutionary entropy will be constrained by the environment in which the protein resides. This constraint will be reflected in structures whose chemically variable parts are situated on the outside of the polymer molecule.

DNA has a unique function, namely, the encoding of genetic information in the sequence of nucleotides. In this case, the maximization of evolutionary entropy will be determined by the internal geometry of the polymer. Maximal evolutionary entropy is achieved by a polymer that forms a double helix, and whose information-containing elements, the nucleotide bases, are located at the core of the helix. This location ensures maximal chemical stability.

#### 1.3.2. DNA and proteins: the origin of age-related diseases

Age-related diseases are the result of irreparable loss in the activity of the biochemical processes that regulate cellular life.

The loss derives from two main developments:

- defects in the replicative process due to mutation in the DNA,
- dysfunction in the metabolic process due to protein misfolding.

The chemical stability of DNA, and the location of the nucleotide bases imply that defects induced by DNA mutations will be uncommon. The interaction of proteins with the external environment indicates that, in spite of the kinetic stability of the polymer, dysregulation of the metabolic machinery will occur. Such defects will be contingent on the external environment, and will be common [2].

These observations entail that age-related familial diseases—disorders induced by DNA mutations—will be rare, and independent of age. However, age-related sporadic diseases—disorders generated by metabolic dysregulation—will be common, with an incidence that increases with age. Studies of the incidence of neurodegenerative diseases such as Alzheimer’s are consistent with these predictions [34].

## 2. The origin of collective behaviour

The physico-chemical principles that underlie the transition from abiotic molecules to an integrated assembly of organic matter are determined by the collective behaviour of the interacting components, inorganic and organic. The collective behaviour and cooperativity of the inorganic components, atoms and molecules that comprise physical systems, are encoded in terms of thermodynamic entropy, a measure of the positional ordering of the microstates that define the macroscopic properties of physical systems.

The collective behaviour and cooperativity of the organic components—activated macromolecules, cells and organisms—that comprise chemical and biological systems are encrypted in terms of evolutionary entropy, a measure of the temporal ordering of the microstates that define the macroscopic properties of these systems.

Statistical thermodynamics exploits the concept of thermodynamic entropy to analyse the collective behaviour of the inorganic components that define physical systems. Directionality theory invokes the concept of evolutionary entropy to study the collective behaviour of the organic components that define chemical and physical systems.

### 2.1. Statistical thermodynamics

Statistical thermodynamics is an analytic theory for explaining how the thermodynamic properties of a macroscopic system arise from interacting microscopic units. A cornerstone of the theory is the concept of thermodynamic entropy. The dynamics of collective behaviour is formalized in terms of the Second Law.

The Second Law loses its validity for small systems, and for systems that are open to the input of energy and matter. Accordingly, the law does not apply to the analysis of collective behaviour of activated macromolecules, cells and higher organisms. Recent applications of statistical thermodynamics to investigate emergent behaviour in biological systems are based on extensions of the Second Law, derivatives of a class of propositions, called fluctuation theorems [35–38].

The fluctuation theorems now constitute a central but controversial element in current efforts to determine the biophysical principles that describe the transition from inorganic matter to cellular life [39–43].

The problematic nature of the thermodynamic formalism resides in two main considerations:

(i) Thermodynamic entropy is not a valid measure of the cooperativity of the components that define organic matter.

Cooperativity between the microscopic components in organic matter involves long-range interactions, and statistical dependencies between the components. Thermodynamic entropy does not incorporate these statistical dependencies, and the temporal progression of microstates, which is a characteristic feature of the collective behaviour of organic matter.

(ii) Heat is not a viable currency for the transfer of information and energy in biological systems.

Energy, the capacity to do work, is expressed in two major forms, potential and kinetic. Potential energy is the energy associated with the position, configuration or shape of an object in a force field. Kinetic energy is the energy associated with a body due to the motion of its component elements.

The forms of energy that drive biological processes are all potential and include [44]:

- the chemical energy contained in the electron bonds that hold atoms together,
- the redox potential manifest during the motion of electrons,
- the photochemical energy that emerges when light is absorbed by the electron structure of a pigment molecule,
- the chemiosmotic energy induced in concentration gradients across membranes.

Thermal energy, the form of energy that plays a critical role in statistical thermodynamics, is random kinetic energy. The maximum work that may be derived from the transfer of heat in a macroscopic system, is given by the expression


$$w=q\;\left(\frac{T_{2}-T_{1}}{T_{2}}\right),$$

where q is the heat absorbed and $T_{2}$ and $T_{1}$ are the absolute temperature of the source and sink [44].

The insignificant nature of the temperature differences in different parts of a cell, the basic unit of living matter, entails that w∼0. Accordingly, thermal energy is not a useful way of affecting the biological work—synthetic, osmotic and muscular—which is necessary to maintain the stability of the living state.

These considerations, the limitations of thermodynamic entropy as a measure of cooperativity, and the isothermal nature of energy transformation in biological systems, are the two main factors that invalidate current efforts to apply the fluctuation theorems of statistical thermodynamics to the study of the origin of life.

### 2.2. Directionality theory

The process of evolution is the adaptive response of a population, by means of variation and natural selection, to the environment in which the population resides. Directionality theory is a mathematical model of the evolutionary dynamics of populations of organic matter. The theory, which has its mathematical roots in the ergodic theory of dynamical systems [45,46], was originally developed to study the evolution of age-structured populations [31,47].

A model of an age-structured population can be described as a strongly connected, directed graph. The nodes of the graph depict age classes, whereas the weighted links between the nodes represent the survivorship rates from one age class to the next and the reproductive rates from the fertile age classes to the class of newborns. The survivorship and the reproductive parameters that are associated with the links of the directed graph correspond to the transfer of energy between age classes.

There exists a unique statistical parameter that quantifies the temporal distribution of energy between the different age classes in the population. This parameter, called evolutionary entropy, describes the rate at which the population converts the chemical energy of the resource into survivorship and offspring production.

In the context of demographic models, evolutionary entropy is a function of the age-specific fecundity and mortality of the individual organisms. Semelparous populations, such as annual plants, have zero entropy. Reproduction in these populations occurs at a single stage in the life cycle. Iteroparous populations, such as perennial plants, distribute their reproductive activity over several age classes. These populations have positive entropy.

The concept of evolutionary entropy has been generalized to incorporate the temporal distribution of energy in populations whose state is parametrized by structural, physiological or behavioural properties [32,33,48].

Evolutionary entropy has now emerged as a generic measure of cooperativity, a parameter that describes the statistical dependencies and correlations induced by cooperative and competitive interactions between the components of a biological network. The statistical measure also describes the rate at which a population converts the chemical energy of resources into biological work.

Directionality theory is the study of dynamical changes in evolutionary entropy induced by variation and natural selection. The principles that determine the adaptive dynamics of evolutionary entropy are contingent on the processes—fluctuation and mutation—that generate variation.

Fluctuation pertains to structural and behavioural variation. These alterations are generated by the lability of the interactions connecting the components. The adaptive dynamics of collective behaviour induced by fluctuations is expressed by the following rule:

*The Entropic Principle of Self-organization*. The collective behaviour of individual components, subject to a self-assembly process of fluctuation and selection, is contingent on the external energy source. The steady states of self-assembly are states that maximize evolutionary entropy.

Mutation refers to genetic variation. These alterations are induced by random changes in the DNA that encode the phenotypic features of the organism.

The adaptive dynamics of collective behaviour driven by a mutation–selection process is formalized by the following rule:

*The Entropic Principle of Evolution.* The collective behaviour of the organic components, subject to the evolutionary process of mutation and natural selection, is contingent on the population size and the external energy source. The steady states of the evolutionary process are extremal states—maxima and minima—of evolutionary entropy.

We will exploit these two principles of variation and selection to generate an adaptive explanation for the emergence of cellular life. We model cellular life as a metabolic-replicating system that involves the dynamic integration of carbon-based polymers:

- *amphiphiles*—these polymers form the boundary membranes of cellular life,
- *proteins*—the catalytic agents,
- *DNA*—the information carriers.

According to the RNA world hypothesis, the transition from inorganic matter to cellular life is characterized by a two-step process:

- the transformation from inorganic matter to protocells—a self-organizing chemical network, involving RNA as an autocatalytic agent
- the transformation from protocells to cellular life—an evolutionary chemical network, organized with proteins as the catalytic agent and DNA as the information carrier.

We will show that the transformation from inorganic matter to protocells can be explained in terms of a sequence of macromolecular transitions, each determined by a fluctuation–selection process and encoded by the *Entropic Principle of Self-organization*.

We will also show that the transformation from protocells to cellular life is elucidated by a sequence of protocellular transitions, each driven by a mutation–selection process and regulated by the *Entropic Principle of Evolution*.

## 3. Evolutionary entropy and Directionality theory

Directionality theory is an analytic model of the collective behaviour of metabolic entities: macromolecules, cells, higher organisms [32,33]. Cooperativity, that is the interaction between the metabolic entities, is described by the statistical parameter, evolutionary entropy.

**Figure 1.**
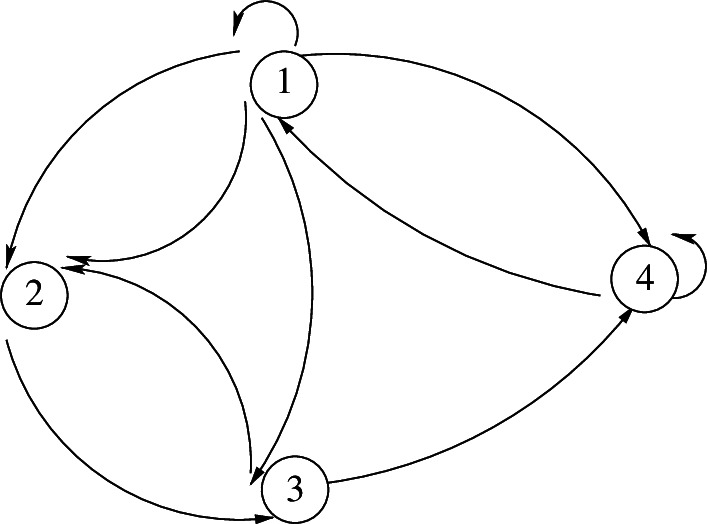
Population as a strongly connected, directed graph.

A network or population, the fundamental unit in models of collective behaviour, is described by a strongly connected, directed graph, as shown in figure 1.

The nodes of the graph represent the microstates of the system, that is the set of physical or chemical properties that describe the metabolic components. The links between the nodes describe the interaction between the components.

We denote the set of nodes in the graph by the set X=(1,2,…d). The model is represented by the mathematical object (Ω,μ,φ), whose elements are defined as follows:

- Ω: the set of all genealogies generated by the interaction between the components. A genealogy is a sequence

$x=(\ldots x_{-1},x_{0},x_{1}\ldots ),$

where $x_{i}$ is an element in the set X.

- φ: a potential function that associates with each element x∈Ω a number that describes the interaction between the components that generate the genealogy.
- μ: a probability measure that describes the distribution of the genealogies. The parameter μ characterizes the steady state of the dynamical system induced by the action of the potential function φ.

Evolutionary entropy denoted by *H,* can be described by the relation *H = S/T* [49], where


(3.1) $$S=-\sum_{\tilde{\alpha }\in C_{\alpha }}p_{\tilde{\alpha }}\;\log p_{\tilde{\alpha }}\;;\;T=\sum_{\tilde{\alpha }\in C_{\alpha }}|\tilde{\alpha }|p_{\tilde{\alpha }}.$$

The element α represents a node of the graph given in figure 1. $C_{\alpha }$ describes the set of trajectories that begin with α, end at α, and do not visit α in the middle. For a given element $\tilde{\alpha }\in C_{\alpha }$ , the quantity $p_{\tilde{\alpha }}$ is the probability of the cycle defined by the trajectory $\tilde{\alpha }$.

Evolutionary entropy, which has its roots in the ergodic theory of dynamical systems, is a measure of the cooperativity of the interacting components that define the network. Cooperativity is described in terms of the number and diversity of the cyclic pathways generated by the interaction of the components.

**Figure 2.**
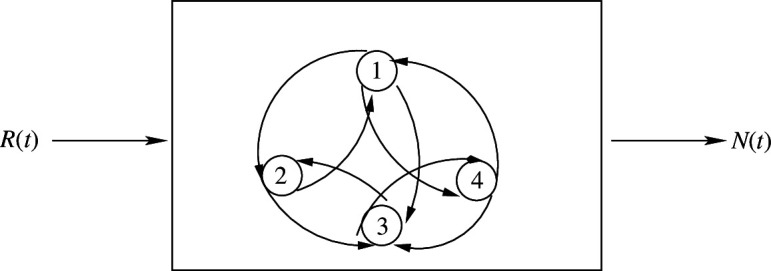
Conversion of the resource endowment R(t) into population numbers N(t).

The model assumes that the population appropriates chemical energy from an external environment, and converts this energy into biological and chemical work. The conversion of chemical energy, in the form of resources R(t), into biological work, in the form of population numbers N(t), is represented in figure 2.

The macroscopic parameters that determine the evolutionary dynamics of the population are determined by the potential φ:Ω→R, and defined as follows [50].

We consider the function


$$S_{n}\varphi (x)=\sum_{k=0}^{n-1}\varphi (\tau^{k}x),$$

where τ denotes the shift operator on Ω


$$\tau :(x_{\kappa })=:({\tilde{x}}_{\kappa }),$$

where $({\tilde{x}}_{\kappa })=x_{\kappa +1}.$

Define


$$Z_{n}(\varphi )=\sum_{\left(x_{0},x_{1},\ldots ,x_{n}\right)}\exp S_{n}\varphi (x^{*}),$$

where for given $\left(x_{0},x_{1},\ldots ,x_{n}\right)$, we denote by $x^{*}$ any point in Ω with $x_{i}^{*}=x_{i}$ for i=0,1,…,n.

The population growth rate, r is given by


(3.2) $$r=\lim_{n\to \infty }\frac{1}{n}\;\log Z_{n}(\varphi ).$$

The demographic variance $\sigma^{2}$, is defined by


(3.3) $$\sigma^{2}=\lim_{n\to \infty }\frac{1}{n}\;{\text{ Var}}_{n}(S_{n}\varphi ).$$

We model the evolutionary process by considering a resident population (Ω,μ,φ), and a variant (Ω,μ(δ),φ(δ)), where φ(δ) is defined by


φ(δ)=φ+δφ,

where δ is a small perturbation.

The dynamics of the incumbent population is determined by the parameters $\left(r,\sigma^{2}\right)$. We assume that the resource production rate, and the variation in the resource production rate are described by the parameters Φ and γ, which are represented as follows:


(3.4) $$\Phi =\frac{dr(\delta )}{d\delta }{|}_{\delta =0}\;;\;\;\gamma =\frac{d\sigma^{2}(\delta )}{d\delta }{|}_{\delta =0}.$$

Directionality theory analyses the dynamics of competition between the incumbent population (Ω,μ,φ) of size N, and the variant population (Ω,μ(δ),φ(δ)) of size $N^{*}$. We will assume $N^{*}\ll N$.

The outcome of competition between the incumbent and the variant population will be determined by the resource endowment, as described by Φ and γ, and the population size M, where $M=N+N^{*}$.

Let y denote the initial frequency of the variant, and P(y) the probability that the variant invades the incumbent.

As shown in [51],


(3.5) $$P(y)=\frac{1-{\left(1-\frac{\Delta \sigma^{2}y}{\sigma^{*2}}\right)}^{\frac{2M_{s}}{\Delta \sigma^{2}}+1}}{1-{\left(1-\frac{\Delta \sigma^{2}}{\sigma^{*2}}\right)}^{\frac{2M_{s}}{\Delta \sigma^{2}}+1}},$$

where s is given by


(3.6) $$s=\Delta r-\frac{1}{M}\Delta \sigma^{2}.$$

The functions Δr and $\Delta \sigma^{2}$ are given by

.$$\Delta r=r^{*}-r\;,\;\Delta \sigma^{2}=\sigma^{*2}-\sigma^{2}$$

The pair $\left(r,\sigma^{2}\right)$ and $\left(r^{*},\sigma^{*2}\right)$ describe the population growth rate and the demographic variance of the resident population and the variant, respectively.

The quantity s determines the convexity of P(y), [51], which we express as follows:


(3.7) s>0⇒P(y) convex; s<0⇒P(y) concave.

The relation between P(y) and s, as described by equation (3.7), indicates that s is a measure of selective advantage, that is, the sign of s determines the outcome of competition between the variant population $X^{*}$ and the incumbent X.

Write


(3.8) $$\tilde{s}=(-\Phi +\gamma /M)\Delta H.$$

As shown in [51],


(3.9) $$s\tilde{s}>0.$$

We conclude from equation (3.8) that the induced change ΔH satisfies the relation


(3.10) (−Φ+γ/M)ΔH>0.

We will illustrate the significance of equation (3.10) by considering the dynamics of natural selection subject to the following two constraints on the variation mechanism:

- *Mutation*—a random change in the informational component of the network.
- *Fluctuation*—a random change in the metabolic component of the network.

### 3.1 Mutation and natural selection

Cellular systems are organized in terms of a genetic and a metabolic component. Variation in cellular systems is generated by mutations, that is changes in the information carrier, DNA. These changes in the genome will induce heritable alterations in cellular behaviour.

The dynamics of collective behaviour at the cellular level thus implicates three processes: mutation—random changes in DNA; inheritance—the transfer of the information from the mother cell to the daughter cells; and selection—competition between the variant and incumbent types for the resource endowment.

Mutation induces an *irreversible* change. We assume that the resident population is described by the demographic parameters $\left(r,\sigma^{2}\right)$, and the variant population by the parameter $\left(r(\delta ),\sigma^{2}(\delta \right)$, and that $\Delta r\neq 0,\;\;\Delta \sigma^{2}\neq 0$.

The resource constraints Φ and γ, as defined by equation (3.4), will therefore satisfy the condition Φ≠0, γ≠0.

We can infer from equation (3.10) that the rules which characterize the change in evolutionary entropy due to mutation and selection are expressed by the following principle:

*The Entropic Principle of Evolution*. The outcome of variation and natural selection on collective behaviour is contingent on the population size, and the external energy source, and characterized by *extremal* states of evolutionary entropy.

The analytic description of the evolutionary principle is the relation [33].


(3.11) (−Φ+γ/M)ΔH≥0.

Here M denotes the total population size.

The relation between the statistical parameters Φ,γ and the directional change ΔH, is summarized in table 1, under the constraint Φγ<0, and table 2, under the constraint Φγ>0.

**Table 1.** Φγ<0: The relation between the statistical parameters Φ and γ, and the directional changes in evolutionary entropy.

constraints on Φ and γ	directional change in evolutionary entropy
Φ<0,γ>0	ΔH>0
Φ>0,γ<0	ΔH<0

**Table 2.** Φγ>0: The relation between the parameters Φ, γ, the population size M, and directional changes in evolutionary entropy.

constraints on Φ,γ, and population size	directional change in evolutionary entropy
Φ<0,γ<0	
γ<MΦ	ΔH>0
γ>MΦ	ΔH<0
Φ>0,γ>0	
γ<MΦ	ΔH<0
γ>MΦ	ΔH>0

Empirical support for the Entropic Principle of Evolution in demographic and metabolic networks is described in Ziehe & Demetrius [52] and Demetrius [33].

### 3.2. Fluctuation and natural selection

Molecular systems are macromolecular structures whose components are held together by non-covalent intermolecular forces. Variation at molecular scales derives from the lability of the interaction connecting the components [53]. In view of this molecular instability entails that chemical bonds may form and break reversibly, thus leading to a continuous reorganization of the components.

The dynamics of collective behaviour will now implicate a fluctuation–selection process. Fluctuation, we recall, is a generic term that describes the changes in structure induced by the lability of molecular interactions.

Fluctuation is a *reversible* process. The resident and variant populations are described by the demographic parameter $\left(r,\sigma^{2}\right)$ and $\left(r(\delta ),\sigma^{2}(\delta )\right)$, respectively.

Since the variation process is driven by the lability of the molecular interaction, $\Delta \sigma^{2}=0$. We conclude from equation (3.4) that γ=0.

Consequently, the changes in evolutionary entropy induced by the fluctuation–selection process will only implicate the parameter Φ. These changes are formalized by the following principle:

*The Entropic Principle of Self-organization*. The outcome of variation and selection on the collective behaviour of the system is contingent on the external energy constraint, and characterized by the *maximization* of evolutionary entropy.

The analytic description of the entropic principle is given by the relation


(3.12) −ΦΔH>0.

Table 3 relates the resource constraints, defined by Φ, with directional changes in evolutionary entropy.

**Table 3.** The relation between the statistical parameter Φ, and directional changes in evolutionary entropy.

constraints on Φ	directional change in evolutionary entropy
Φ<0	ΔH>0
Φ>0	ΔH<0

The relation given by (3.12) is a general rule for the emergence of self-organization. This rule pertains to two processes of self-assembly: static and dynamic. The distinction between these two modes of self-assembly is based on the following identity:


(3.13) r=H+Φ.

This relates the population growth rate r, with the evolutionary entropy H and the reproductive potential Φ.

Static self-assembly describes processes in which there is no dissipation of energy. These systems satisfy the relation r=0. We conclude from equation (3.13) that static self-assembly satisfies the condition H+Φ=0.

In view of equation (3.12), we infer that, under static self-assembly, we have the relation


(3.14) ΔH≥0.

The relation (3.14) entails the following principle:

*Entropic Principle of static Self-assembly*. The equilibrium states for the process of static self-assembly are states that maximize evolutionary entropy.

Dynamic self-assembly processes are characterized by the condition r≠0. We can invoke (3.12) and (3.13) to show that dynamic self-assembly implies the relation


(3.15) (H−r)ΔH≥0.

The condition (3.15) yields the following principle:

*The Entropic Principle of dynamic Self-assembly*. The equilibrium state of dynamic self-assembly is a steady state that maximizes evolutionary entropy, contingent on energetic constraints.

## 4. The emergence of cellular life

The transformation from inorganic matter to the emergence of chemical assemblies capable of evolution by variation and natural selection can be depicted in terms of the following two processes:

(I) *Inorganic matter to protocells.* The emergence of the protocellular state can be depicted in terms of the following transitions:

- *Amphiphiles to vesicular polymers.* The polymerization of amphiphilic molecules to form stable vesicles.
- *Nucleic acids to informational polymers: the RNA world.* The polymerization of nucleic acids to generate RNA with catalytic and informational capabilities.
- *The emergence of protocells: The transformation to the protocellular state*. Protocells are characterized by a membrane that defines a spatially localized compartment, and an informational polymer that enables the replication and inheritance of functional information.

We will show that the transitions in Phase (I) can be explained in terms of the *Entropic Principle of Self-organization*.

(II) *Protocells to cellular life.* Protocells are a highly elaborate set of self-reproducing chemical reactions that rely exclusively on RNA for catalysis and replication.

The emergence of cellular life from the protocellular state can be described in terms of the following two transitions:

- The transition to cells, defined by an assembly of RNA and protein—the evolution of protein catalysts.
- The transition to cells, defined by an assembly of DNA–RNA and protein—the evolution of DNA replication.

We will show that the transitions in Phase (II) can be explained in terms of the *Entropic Principle of Evolution.*

### 4.1. Inorganic matter to protocells

Geological and geophysical evidence indicates that the Earth’s atmosphere was once reducing and composed predominantly of the inorganic states of matter: methane, nitrogen and ammonia. The transition from inorganic matter to protocells, the fundamental unit of cellular life, can be described in terms of the following sequence of transformations.

#### 4.1.1. Inorganic matter to activated biomolecules (*dynamic self-assembly*)

Experimental studies indicate that organic molecules can be created without the mediation of enzymes [9]. The transition is driven by an external energy source—heat and electrical discharge.

The theoretical basis for the transition is a model where the initial state is a random assembly of inorganic molecules. Evolutionary entropy, a measure of the cooperativity of the interacting components, will be small.

We now assume that the system is furnished with a constant supply of energy in the form of electric discharges. The effect of this constant source of energy is the generation of non-covalent interactions between the inorganic molecules, and the emergence of components with differences in cooperativity, consequently, differences in evolutionary entropy. Competition between the local structures for the energy source entails that intermediates with increased evolutionary entropy, and concomitantly, increased stability will emerge. The increase in evolutionary entropy will continue until a value is attained that exceeds the growth rate r. The critical level of organization in the model is described by an evolutionary entropy that satisfies the condition,

.H>r

#### 4.1.2. Activated biomolecules to amphiphilic molecules (*static self-assembly*)

The chemical reactions that define replication and metabolism take place in membrane-bounded compartments. The membranes consist of polymers, composed of organic components called amphiphiles.

Experimental studies indicate that lipid vesicles self-assemble when a phospholipid is dispersed in aqueous solution [30]. The self-assembly of membranous vesicles is a spontaneous process. This ordering does not require a constant source of energy. Self-organization in these systems is defined by static self-assembly. The equilibrium state is given by a configuration that maximizes evolutionary entropy.

.ΔH>0

#### 4.1.3. Activated biomolecules to informational polymers (*dynamic self-assembly*)

Modern biology is constructed from a large set of chemical reactions defined by replicating DNA and catalytic proteins.

The complexity of DNA and protein polymers suggests that these systems are the derivatives of RNA polymers, the fundamental units of the RNA world.

The chemical bonds linking monomers into polymers are produced by the removal of water molecules. This chemical reaction can only proceed with input from an external energy source. Accordingly, the transition from activated nucleotide monomers to RNA polymers can be described as dynamic self-assembly. Accordingly, the equilibrium configuration will satisfy the relation,

.(H−r)ΔH≥0

### 4.2. Protocells to cellular life

A protocell consists of two key components: a protocell membrane that defines a spatially localized compartment, and an informational polymer consisting of RNA molecules capable of replication and catalysis.

Modern cellular life is the sequential assembly of three classes of carbon-based polymers [29].

- *Amphiphilic molecules*. These are components, phospholipids, fatty acids and sterols, that contain covalently bonded components, with one part having a high affinity for polar solvents, and the other part a high affinity for non-polar solvents. These molecules are permeability barriers so that modern cells have complete control over the uptake of nutrients and the export of wastes.
- *Proteins*. These carbon-based polymers are chains of amino acids. Proteins are characterized by their capacity to fold into three-dimensional structures with catalytic activity.
- *DNA*. These polymers store and replicate genetic information. The major difference between DNA and RNA is the presence of a hydroxyl group in RNA, a property that makes RNA less stable.

The transition from an RNA world, defined by RNA polymers, to cellular life, defined by the replacement of RNA by protein and DNA, can be explained in terms of the *Entropic Principle of Evolution*.

**Figure 3.**
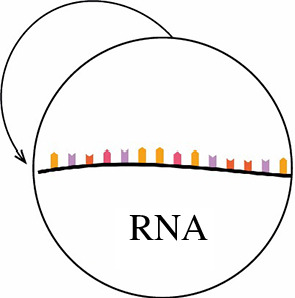
RNA–based protocells.

The three processes that define the transition from an RNA world to a system involving RNA, proteins and DNA can be described by the following processes.

(I) *The transition RNA → RNA* figure 3.

RNA in protocells functions as inefficient catalysts and as unstable information carriers.

**Figure 4.**
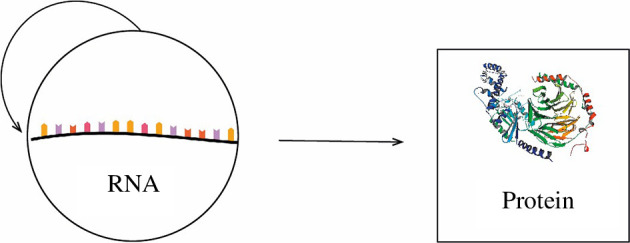
The transition RNA → proteins.

RNA differs from proteins in terms of size and the capacity to form complex structures. These features impair its catalytic effectiveness. RNA differs from DNA in having an extra hydroxyl group on each of its sugars. This property enhances specific catalytic activity, but increases reactivity and the capacity to store genetic information [2] .

(II) *The transition RNA* → *proteins* figure 4.

**Figure 5.**
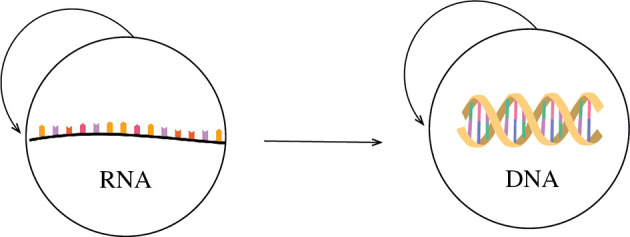
The transition RNA → DNA.

Protein synthesis is assumed to have evolved from RNA-based protocells. The transition from RNA to proteins confers a selective advantage on account of the superior catalytic activity of proteins.

(III) *The transition RNA → DNA* figure 5*.*

Modern DNA replication occurs by a mechanism that involves the opening of the parental double helix into two separate strands so that each strand can serve as a template for the formation of a new strand. The stability of DNA entails that the replacement of RNA by DNA confers a selective advantage, as it ensures a low error rate and fidelity of the information transfer process [2].

The transitions RNA → Protein and RNA → DNA can be explained in terms of the *Entropic Principle of Evolution*. The selective advantage conferred by the transition RNA → protein is the enhancement of catalytic activity. The selective advantage induced by the transition RNA → DNA is the increase in the stability of the genome.

## 5. Conclusion

The fundamental units of inorganic matter are atoms and molecules. The collective behaviour and cooperativity of these elements define the properties of solids, liquids and gases—the entities that constitute condensed matter.

The *Second Law of Thermodynamics* pertains to the conversion of thermal energy into mechanical work. The law is expressed in terms of the statistical parameter *thermodynamic entropy,* a measure of the positional ordering of the interacting components of the system. This ordering ignores the statistical dependencies and correlations that may arise from long-range interactions between the components. *The Second Law* asserts that the collective behaviour of systems that are isolated and closed to the input of energy and matter is characterized by states that maximize thermodynamic entropy.

The fundamental units of living matter are protocells, metabolic units defined by a spatially localized compartment enclosing the carbon-based polymers: DNA, RNA and proteins. The collective behaviour and cooperativity of these units determine the evolution of protocells, the progenitor of cellular life.

The *Entropic Principle of Evolution,* which pertains to energy transformation in biological systems, encodes the conversion of the chemical energy of resources into biological work. The principle is expressed in terms of *evolutionary entropy,* a measure of the temporal ordering of the interacting components. This ordering incorporates the statistical dependences, and the correlations that may arise from the long-range interaction between the components of the system. *The Entropic Principle* asserts that the collective behaviour of systems that are driven by an external energy source is contingent on the resource endowment and determined by extremal states of evolutionary entropy.

**Figure 6.**
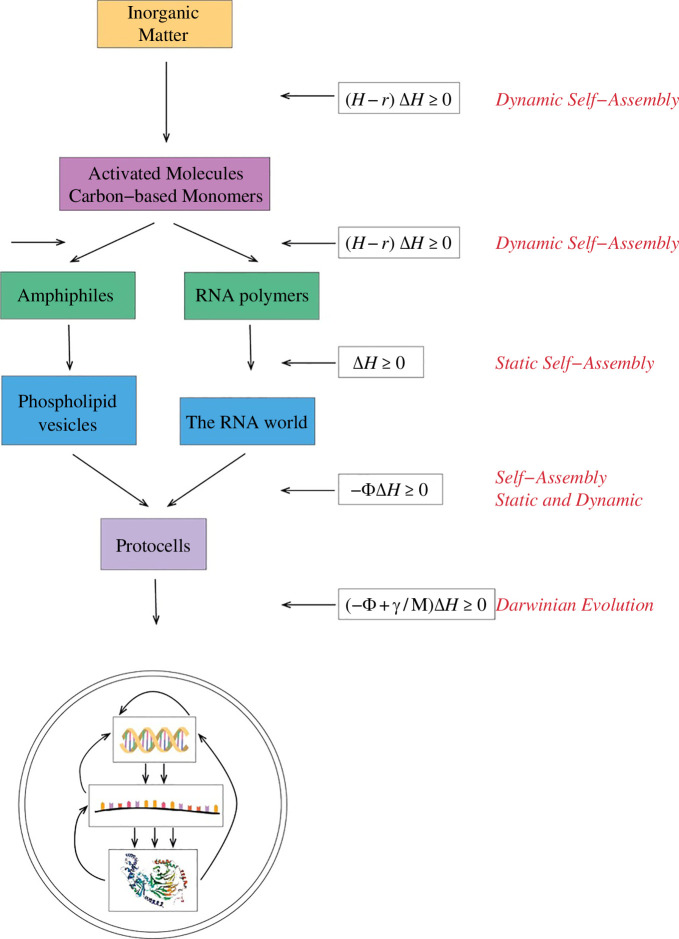
The transition from inorganic matter to cellular life.

Figure 6 presents a graphic description of the sequence of steps that mark the transition from inanimate matter, whose collective behaviour is prescribed by the *Second Law,* to cellular life, whose collective behaviour is regulated by the *Entropic Principle of Evolution*.

We will now provide an analytic complement to figure 6 by describing the adaptive principles that regulate the transition from the process, regulated by the *Second Law of Thermodynamics,* to the sequence of processes determined by the *Entropic Principle of Evolution*.

### 5.1. Collective behaviour: Statistical Thermodynamics

The collective behaviour of the individual components of inanimate matter is based on the following assumptions:

I(a) The particles that comprise the aggregate are identical.
I(b) The number of individual components is very large.
I(c) The evolution of the system is governed by Hamiltonian dynamics.
I(d) The system is isolated, and closed to the input of energy and matter.

The model proposed by Boltzmann, analyses the evolution of collective behaviour by assigning to each microstate of the system, a number, the thermodynamic entropy $S_{B}$, given by


(5.1) $$S_{B}=k\log W.$$

The quantity W describes the total number of microstates of a system consistent with a given macrostate.

If we assume that the particles in the macroscopic system occupy small volumetric cells, i=1,2,…s of phase space, with occupation numbers $n_{i}$, and that the total number N of particles is conserved, the thermodynamic entropy $S_{B}$ can be expressed by


(5.2) $$S_{B}=-k\sum_{j}p_{j}\;\log p_{j},$$

where $p_{j}=\frac{n_{j}}{N}.$

In systems closed to the input of energy and matter, the collective behaviour of the macroscopic system satisfies the equation


(5.3) $$\Delta S_{B}>0.$$

### 5.2. Collective behaviour: Directionality theory

The collective behaviour of aggregates of organic matter—activated macromolecules, cells and higher organisms—is distinguished from inorganic matter in terms of the size of the system, the long-range nature of the interaction between the components, and the existence of an external energy source.

The model is based on the following assumptions:

II(a) The individual components that define the system are distinct.
II(b) The number of components is finite.
II(c) The collective behaviour of the system is driven by a process of variation and selection.
II(d) The system is driven by an external source of energy.

The model of the collective behaviour of this process assigns to the network of interacting components, a measure of cooperativity, called evolutionary entropy, denoted H and given by H=S/T.

The parameter S, which we also call evolutionary entropy, and T, the cycle time, are given by [33]


(5.4) $$S=-\sum_{\tilde{\alpha }\in C_{\alpha }}p_{\tilde{\alpha }}\log p_{\tilde{\alpha }};\;\;T=\sum_{\tilde{\alpha }\in C_{\alpha }}|\tilde{\alpha }|p_{\tilde{\alpha }}.$$

The element α denotes a node of the directed graph that describes the network. $C_{\alpha }$ denotes the set of all genealogies that begin with α, and end with α without visiting α inbetween.

For a given element $\tilde{\alpha }\in C_{\alpha }$, the quantity $p_{\tilde{\alpha }}$ is the probability of the cycle defined by the genealogy $\tilde{\alpha }$.

This measure of cooperativity of the biological network will evolve as new variants are introduced in the population and compete with the resident population for resources. The change ΔH in evolutionary entropy, induced by this process of variation and selection, satisfies the equation


(5.5) (−Φ+γ/M)ΔH≥0.

The statistical parameters, Φ and γ, are correlated with the dynamics of the resource endowment, a property of the external environment. The parameter M is the population size.

### 5.3. The Entropic Principle of Evolution and the Second Law of Thermodynamics

We will elucidate the relation between the Second Law of Thermodynamics, as expressed by equation (5.3), and the Entropic Principle of Evolution, as illustrated by equation (5.5), by considering various constraints on the resource parameters Φ and γ, and the population growth rate r.

We recall that the statistical parameters Φ and γ satisfy the relation

.$$\begin{array}{c} \Phi =\frac{dr(\delta )}{d\delta }{|}_{\delta =0}\;;\gamma =\frac{d\sigma^{2}(\delta )}{d\delta }{|}_{\delta =0} \end{array}$$

We note that the population growth rate, r, satisfies the relation

(5.6) r=H+Φ.

So we now impose the following set of constraints on the statistical parameters.

(I) *The constraintγ=0*

This constraint on γ implies that the mechanism that regulates the process of variation is a *fluctuation*—a change induced by the lability of the components. The condition γ=0 implies that the relation (5.5) reduces to


(5.7) −ΦΔH>0.

This relation describes the *Entropic Principle of Self-organization*: The outcome of the fluctuation–selection process is contingent on the external energy source, and characterized by the maximization of evolutionary entropy.

(II) *The constraintr=0*

This condition describes a system that is closed to the input of energy and matter.

This constraint on the growth rate r yields the relation


(5.8) ΔH≥0.

This relation describes the *Entropic Principle of static Self-assembly*: The outcome of the fluctuation–selection process is characterized by states that maximize evolutionary entropy.

(III) *The condition N→∞, where N denotes the number of components*

As N→∞


(5.9) $$\Delta S_{B}.\Delta S\geq 0,$$

where S, the evolutionary entropy, is given by equation (5.4).

Since

,ΔS.ΔH>0

we infer from equation (5.9) that, as N→∞


(5.10) $$\Delta H.\Delta S_{B}\geq 0.$$

The relation between the *Entropic Principle of Evolution* and the *Second Law,* as described by (5.11), is the analytic complement to the biomolecular and protocellular transitions described in figure 6,


(5.11) $$(-\Phi +\gamma /M)\Delta H\geq 0\;\;\overset{\gamma =0}{⟶}\;\;(-\Phi )\Delta H\geq 0\;\overset{r=0}{⟶}\;\;\Delta H\geq 0\;\;\;\overset{N\to \infty }{⟶}\;\;\Delta S_{B}>0.$$

The first relation in (5.11) encodes the *Entropic Principle of Evolution,* the rule that characterizes the transition from protocells to cellular life. The second and third relations formalize the *Entropic Principle of Self-Organization*, the rules of self-assembly that encode the transformation from activated macromolecules to protocells. The fourth relation depicts the *Second Law*.

## Data Availability

This article has no additional data.
